# Low-Dose Pesticides Alter Primary Human Bone Marrow Mesenchymal Stem/Stromal Cells through ALDH2 Inhibition

**DOI:** 10.3390/cancers13225699

**Published:** 2021-11-14

**Authors:** Amélie Foucault, Noémie Ravalet, Joevin Besombes, Frédéric Picou, Nathalie Gallay, Laetitia Babin, Jérôme Bourgeais, Sophie Hamard, Jorge Domenech, Pascal Loyer, Nicolas Vallet, Julien Lejeune, Emmanuel Gyan, Marie C. Béné, François Vallette, Christophe Olivier, Olivier Hérault

**Affiliations:** 1 Department of Biological Hematology, Tours University Hospital, 37000 Tours, France; amelie.foucault@univ-tours.fr (A.F.); noemie.ravalet@univ-tours.fr (N.R.); joevin.besombes@etu.univ-tours.fr (J.B.); f.picou@chu-tours.fr (F.P.); n.langonne@chu-tours.fr (N.G.); j.bourgeais@chu-tours.fr (J.B.); jorge.domenech@univ-tours.fr (J.D.); 2 CNRS ERL 7001 LNOx, EA 7501, Tours University, 37000 Tours, France; laetitia.babin@univ-tours.fr (L.B.); sophie.hamard@univ-tours.fr (S.H.); julien.lejeune@univ-tours.fr (J.L.); emmanuel.gyan@univ-tours.fr (E.G.); 3 INSERM U1241, NuMeCan Institute, 35000 Rennes, France; pascal.loyer@univ-rennes1.fr; 4 CNRS GDR 3697 Micronit “Microenvironment of Tumor Niches”, 37000 Tours, France; francois.vallette@inserm.fr; 5 Cancéropole Grand Ouest (CGO), NET “Niches and Epigenetics of Tumors” Network, 44000 Nantes, France; 6 Department of Hematology & Cell Therapy, Tours University Hospital, 37000 Tours, France; nicolas.vallet@inserm.fr; 7 Department of Biological Hematology & CRCINA, Nantes University Hospital, 44000 Nantes, France; mariechristine.bene@chu-nantes.fr; 8 Fédération Hospitalo-Universitaire, “Grand Ouest Against Leukemia” (FHU GOAL), 49000 Angers, France; 9 INSERM UMR 1232 CRCINA, 44000 Nantes, France; christophe.olivier@univ-nantes.fr; 10 Faculty of Pharmaceutical and Biological Sciences, Nantes University, 44000 Nantes, France; 11 OPALE Carnot Institute, The Organization for Partnerships in Leukemia, Hôpital Saint-Louis, 75010 Paris, France

**Keywords:** hematopoiesis, pesticides, mesenchymal stem/stromal cells, ALDH2, myelodysplastic syndrome

## Abstract

**Simple Summary:**

Pesticide exposure is a public health concern. Two recent studies reported that exposure of normal primary bone marrow mesenchymal stem/stromal cells (BM-MSCs) to low-dose pesticide mixture induces deleterious consequences, such as oxidative stress, senescence, accelerated aging and increased sensitivity to oncogenic hits. Here, we show that exposure to this mixture decreases aldehyde dehydrogenase-2 (ALDH2) activity, with a concomitant increase in acetaldehyde level and DNA damage and alters the MSC capacity to support primitive hematopoiesis. Similar abnormalities were observed in bone marrow-MSCs from patients suffering from myelodysplastic syndrome (MDS), suggesting a role of pesticide-induced ALDH2 inhibition in the pathophysiology of this pre-leukemic disease.

**Abstract:**

(1) Background: The impact of occupational exposure to high doses of pesticides on hematologic disorders is widely studied. Yet, lifelong exposure to low doses of pesticides, and more particularly their cocktail effect, although poorly known, could also participate to the development of such hematological diseases as myelodysplastic syndrome (MDS) in elderly patients. (2) Methods: In this study, a cocktail of seven pesticides frequently present in water and food (maneb, mancozeb, iprodione, imazalil, chlorpyrifos ethyl, diazinon and dimethoate), as determined by the European Food Safety Authority, were selected. Their in vitro effects at low-doses on primary BM-MSCs from healthy volunteers were examined. (3) Results: Exposure of normal BM-MSCs to pesticides for 21 days inhibited cell proliferation and promoted DNA damage and senescence. Concomitantly, these cells presented a decrease in aldehyde dehydrogenase 2 (ALDH2: mRNA, protein and enzymatic activity) and an increase in acetaldehyde levels. Pharmacological inhibition of ALDH2 with disulfiram recapitulated the alterations induced by exposure to low doses of pesticides. Moreover, BM-MSCs capacity to support primitive hematopoiesis was significantly altered. Similar biological abnormalities were found in primary BM-MSCs derived from MDS patients. (4) Conclusions: these results suggest that ALDH2 could participate in the pathophysiology of MDS in elderly people long exposed to low doses of pesticides.

## 1. Introduction

The increasing usage of pesticides worldwide [1], and its consequences on human health are an important public concern. Many research projects have been conducted to clarify the carcinogenic risk in case of occupational pesticide exposure (OPE). The association between OPE and hematological malignancies has been well established for lymphoid diseases several years ago [2]. A recent report of French Institute of Health and Medical Research (INSERM) reviewed the pesticides for which there is a strong presumption of a link between bone marrow disease (multiple myeloma) and exposure to high doses of pesticides [3]. Pesticides reported are dichlorodiphenyltrichloroethane (DDT, organochlorine family), carbaryl (carbamate family), permethrin (pyrethroid family), glyphosate (aminophosphonate glycine) and captan (phthalimide family). These pesticides can affect the human bone marrow, but it is a non-exhaustive list since all medical studies concern exposure to high doses of pesticides.

This association between OPE and hematological malignancies was suspected for myelodysplastic syndrome (MDS), a preleukemic disease [4,5]. Moreover, our group has recently published a meta-analysis reporting an increased risk of acute myeloid leukemia (AML) occurrence after exposure to high-dose pesticides [6]. Little is known however regarding the potentially deleterious effects on hematopoiesis of long-term exposure to low-dose pesticides, and to date, no hematotoxicity was reported. This question is of interest since the general population is exposed daily to pesticide residues, especially due to the contamination of water, fruits and vegetables [7]. Specific institutions such as the European Food Safety Authority (EFSA) monitor pesticide residues found in food to determine the acceptable daily intake (ADI) of each of them. Unfortunately, these data do not consider the cocktail effect of pesticides mixtures, which could potentially increase the dangerousness of these xenobiotics [8]. According to a recent EFSA report, pesticide residues were found in 41% of analyzed food samples [8].

It may thus be hypothesized, in light of alterations reported for high-dose OPE, that hematopoiesis could also be altered by lifelong exposure to low doses of such cocktails, notably via the long-term impregnation of the microenvironment by low-dose pesticides, which could participate in the dysregulation of normal hematopoiesis and induce myeloid malignancies. This postulate is reinforced by recent publications of our research consortium, in which BM-MSCs exposed to a cocktail of low doses of the seven pesticides frequently identified in the last few years in food samples (maneb, mancozeb, iprodione, imazalil, chlorpyrifos ethyl, diazinon, dimethoate, classified by decreasing abundance in analyzed samples [8]) presented such functional alterations as a decrease in proliferation, an adipocytic differentiation bias, the induction of oxidative stress, the promotion of senescence and an increased sensitivity to oncogenic hits [9,10].

The bone marrow (BM) microenvironment and more particularly BM-MSCs play critical roles in sustaining normal hematopoiesis. Alterations of these cells are also involved in the pathophysiology of several types of malignant hematological disorders. In MDS and AML, a decrease in the proliferative capacity of BM-MSCs has been shown to be concomitant to normal hematopoiesis failure [11]. Moreover, BM-MSCs from AML patients have been reported to express early adipocyte differentiation markers and an aberrant DNA methylation profile [11,12,13,14,15]. Several studies further suggest that alterations of BM-MSCs can lead to the appearance of malignant clones in vivo [14,15,16,17]. Myeloproliferative disorders have been reported after loss of expression of the retinoic acid receptor-gamma (RARγ) by BM-MSCs in an altered murine microenvironment [16]. MDS features have appeared in mice invalidated for *Dicer1* gene in the osteoblastic lineage (Dicer1^lox/lox^-Osterix-Cre) [17]. AML have developed in mice following constitutive activation of the Wnt pathway, FoxO1/activated β-catenin interaction or a genetic alteration of the Notch pathway in the osteoblastic lineage [18,19].

Here, we examined the effect of the cocktail of seven pesticides mentioned above, at low-doses, on primary normal BM-MSCs and compared them to the features of BM-MSCs from MDS patients. We confirmed the appearance of a high level of reactive oxygen species (ROS), senescence and DNA damage. We then demonstrated that these alterations are related to an inhibition of ALDH2. Finally, we observed that the inability of primary MDS BM-MSC to sustain primitive hematopoiesis, which present similar alterations of the ALDH2 axis, was reproduced in normal BM-MSCs exposed to low-dose of pesticides.

## 2. Materials and Methods

### 2.1. Cells

Primary bone marrow MSCs (BM-MSCs) were isolated and characterized functionally as reported previously [20] from young healthy volunteers (HEALTHOX protocol; Clinicaltrials.gov # NCT02789839, University Hospital, Tours, France) and MDS patients (MDS-BM-MSCs) (MYLESYM protocol, CPP Tours and AFSSAPS: 2011-A00262-39) (Table S1). Normal CD34^+^ cells used for cobblestone area forming cell (CAFC) assays were obtained during peripheral collection after G-CSF stimulation. CD34^+^ cells were enriched using a magnetic bead separation kit (Mini-MACS^®^, Miltenyi Biotec, Gladbach, Germany), as described [21] and the purity of selected cells was >85%. Cell viability in all experiments was quantified by trypan blue exclusion.

### 2.2. Cell Culture and Pesticide Treatment

Three doses of pesticides were used. The first was the high nutritional daily intake (hNDI), calculated for the French population of all ages and corresponding to the lowest doses of pesticides used in our study. The second was the ADI, which is the threshold of safety in humans for lifetime exposure. The last was a value of three times the ADI (3ADI).

BM-MSCs were amplified as previously described [20]. Briefly, they were cultured in 150 cm^2^ flasks (Corning^®^ cell culture flask, Sigma-Aldrich, St. Louis, MO, USA) for 3 weeks (37 °C, 5% CO_2_) in α-MEM supplemented with FGF2 (1 ng/mL, R&D system, Minneapolis, MN, USA) and were tested at passages 2 to 4 (2000–4000 cells/cm^2^). This medium, supplemented with pesticides (hNDI, ADI and 3ADI), was renewed every 3 days. The doses of pesticides used (chlorpyrifos ethyl, dimethoate, diazinon, iprodione, imazalil, maneb and mancozeb; Sigma-Aldrich) were extrapolated, for each pesticide, from 3 values according to previous studies (Table S2) [9,10].

### 2.3. RNA Extraction and Quantitative Reverse Transcription PCR (RT-qPCR)

After 21 days of pesticide exposure, BM-MSCs mRNA was extracted and ALDH2 transcripts were quantified by RT-qPCR as previously described [22]. The geometric Ct means of human *ACTB*, *B2M* and *GAPDH* genes were used to normalize the expression of ALDH2 transcripts [23]. ALDH2—F: cctctatgtggccaacctg, R: ccaaatccaggcacaatgtt and probe: cagcctcc; ACTB—F: attggcaatgagcggttc, R: cgtggatgccacaggact and probe: gctggaag; B2M—F: ttctggcctgcaggctatc, R: tcagcaaatttgactttacattc and probe: ccagccgc; GAPDH—F: agccacatcgctcagacac, R: gcccaatacgaccaaatcc and probe: ccagccgc.

### 2.4. ALDH Family Expression Array

cDNAs were synthesized using the RT2 First Strand Kit (Qiagen, Hilden, Germany) from 500 ng total RNA, and qPCR reactions were performed using the Custom RT2 PCR Array Kit (Qiagen), following the supplier’s guidelines.

### 2.5. Protein Extraction and Western Blot Analyse

After 21 days of pesticide exposure, BM-MSCs were lysed in NP40 cell lysis buffer (Invitrogen by ThermoFisher Scientific, Waltham, MA, USA) containing protease and phosphatase inhibitors. Western blot analyses were performed using rabbit polyclonal antibodies against human ALDH2 diluted at 1/1000 (Proteintech, Rosemount, IL, USA) and HRP-conjugated goat anti-rabbit IgG antibody (Vector Laboratories, Burlingame, CA, USA) at 1/10,000. Detection was performed with ChemiDocTM Gel Imaging System (Bio-Rad Laboratories, Redmond, WA, USA). 

### 2.6. ALDH2 Activity, Acetate and Acetaldehyde Levels

ALDH2 activity was measured using the mitochondrial aldehyde dehydrogenase activity assay kit (Abcam, Cambridge, UK). Acetate concentration was determined with the acetate assay kit (Abcam) following the supplier’s guidelines. Acetaldehyde concentration was quantified as following: (i) sonication of 10^7^ cells in PBS; (ii) centrifugation at 10,000× *g*, 20 min, 4 °C and collection of supernatants; (iii) quantification of acetaldehyde concentration with the acetaldehyde assay kit (Megazyme, Bray, Ireland) following the supplier’s guidelines. Optical density was measured with CLARIOstar^®^ monochromator microplate reader (BMG Labtech, Offenburg, Germany).

### 2.7. Senescence Analysis

Senescent cells were quantified by using a histochemical staining kit (SA-beta-Galactosidase, Sigma-Aldrich) following the supplier’s guidelines. Cells were fixed with 4% paraformaldehyde for 15 min, permeabilized (PBS/Triton X-100 0.1%, 10 min, room temperature), blocked with gelatin from cold water fisher skin 5% (Sigma-Aldrich) Triton X-100 0.1% (Sigma-Aldrich) in PBS and were incubated overnight at 4° C with anti-GLB1/ß galactosidase polyclonal rabbit antibody (Abcam) at 1/500, followed by secondary anti-rabbit labeled with FITC (Jackson Immunoresearch, Cambridge, UK) at 1/2000 at room temperature for 2 h.

### 2.8. Comet Assay

DNA fragmentation was measured by alkaline comet assay after pesticides exposure. Briefly, 70,000 cells were seeded on low melting point agarose (Trevigen, Gaithersburg, MD, USA). CometSlide (Trevigen) was filled with 70 μL of the cell/agarose suspension and was placed at 4 °C for 5 min. The cells were then lysed and left at 4 °C for 30 min, transferred to freshly prepared cold electrophoresis buffer (200 mM NaOH, 1 mM EDTA) and incubated at room temperature in the dark for 40 min. Then, electrophoresis was performed in the CometAssay^®^ Electrophoresis System (Trevigen) at 21 V for 30 min. Slides were washed and fixed (5 min in 70% EtOH), dried and stained 5 min at 4 °C with SYBR Green I (Trevigen) or DAPI (Invitrogen) diluted 1/10,000. Fluorescence images were acquired with a Ti Series Nikon eclipse (Nikon, Tokyo, Japan) and analyzed using ImageJ software version 1.53 (Wayne Rasband, NIH, MA, USA).

### 2.9. Phospho-S139 γH2AX Quantification by Flow Cytometry

Phospho-S139 γH2AX quantification was performed by flow cytometry as previously described [24] using FITC-conjugated anti-phospho-S139 γH2AX antibody (Millipore, Burlington, MA). Analyses were performed with BD Accuri C6^®^ (Becton-Dickinson, Franklin Lakes, NJ, USA). Data were analyzed using FlowJo^®^ v10.1 software (FlowJo LLC, Ashland, OR, USA).

### 2.10. Foci Analyses by Immunofluorescence

After 21 days, cells were fixed with 4% paraformaldehyde, permeabilized (PBS/Triton X-100 0.1%, 10 min, room temperature), blocked with gelatin from cold water fish skin 5%, Triton X-100 0.1% (Sigma-Aldrich) in PBS, and were incubated overnight at 4 °C with anti-phospho-S139 γH2AX monoclonal mouse antibody (Abcam) at 1/1000, anti-MDC1 polyclonal rabbit antibody (Novus Biologicals, Centennial, CO, USA) at 1/500 and anti-53BP1 polyclonal goat antibody (R&D systems) at 1/1000, followed by a secondary anti-mouse antibody labeled with TRITC (Jackson Immunoresearch, Ely, UK), Alexa Fluor^®^ 647 conjugated anti-goat (Jackson Immunoresearch) and FITC-conjugated anti-rabbit (Jackson Immunoresearch) at 1/2000 each at room temperature for 2 h. Fluorescence images were acquired with a Leica TCS SP8 (Leica, Wetzlar, Germany) confocal microscope and analyzed using ImageJ software.

### 2.11. Limiting Dilution CAFC Assay

BM-MSCs were exposed to pesticides only during the 21 days of expansion culture before co-cultures with normal CD34^+^ cells, and CAFC assays were performed as previously described and as detailed in supplementary methods [22]. Briefly, CD34^+^ cells were thus co-cultured with the pesticide-exposed BM-MSCs for 7 days (37 °C, 5% CO_2_), seeded at 6000 CD34^+^/cm^2^ (ratio CD34^+^ cells/BM-MSCs = 1/3) in Myelocult™ H5100 medium (StemCell Technologies, Vancouver, Canada). At the end of coculture, CD34^+^ cells were sorted (BD FACSMelody^TM^ cell sorter, Becton-Dickinson) and cultured on MS5 stromal cells [25] for 5 additional weeks in 96-well plates (37 °C, 5% CO_2_) at 6 different dilutions (range 1–100 CD34^+^ cells/well) in 0.2 mL of Myelocult™ H5100 medium. After 5 weeks of co-culture, each well was examined by inverted phase contrast microscopy (DMI 3000 B, Leica) to identify cobblestone areas. The percentage of wells with at least one CAFC was quantified and the frequency of CAFC in the initial CD34^+^ cell population was calculated as previously described [26]. The CAFC proliferative capacity was determined by subculturing the wells containing one cobblestone area in semi-solid medium (MethoCult^TM^ H4434, StemCell Technologies) to determine the mean total number of clonogenic progenitors (including CFU-GM, BFU-E and CFU-M) generated by each CAFC.

### 2.12. ALDH2 Inactivation

Crispr/Cas9 knockdown of *ALDH2* gene expression was performed with guide RNA (gRNA) targeting the *ALDH2* gene (or scramble gRNA). Crispr/Cas9 knockdown of *ALDH2* gene expression was designed using CRISPOR software version 4.97 [27] or based on previously published data [28]. Human primary BM-MSCs were transfected using TransIT-CRISPR^®^ Transfection Reagent (Sigma-Aldrich). ALDH2 mRNA and protein levels were quantified as described above.

ALDH2 inhibition in BM-MSCs by disulfiram [29] was performed by exposure of BM-MSCs (cultured in 75 cm^2^ flasks as previously described) for two weeks to 0.05 µM and 0.1 µM disulfiram.

### 2.13. Statistical Analyses

Results are expressed as mean ± standard error of the mean (SEM). Statistical analyses were performed on non-normalized data using Friedman, Mann–Whitney or Kruskall–Wallis non-parametric tests and post hoc Dunn’s multiple comparisons test. Statistics were performed with GraphPad PrismVR version 7.02 or R version 3.6.3 (https://www.r-project.org/, accessed on 29 March 2021). Differences with *p* < 0.05 were considered statistically significant (NS: not significant).

## 3. Results

### 3.1. Pesticide Exposure Induces DNA Damage in Normal BM-MSCs

**Figure 1:**
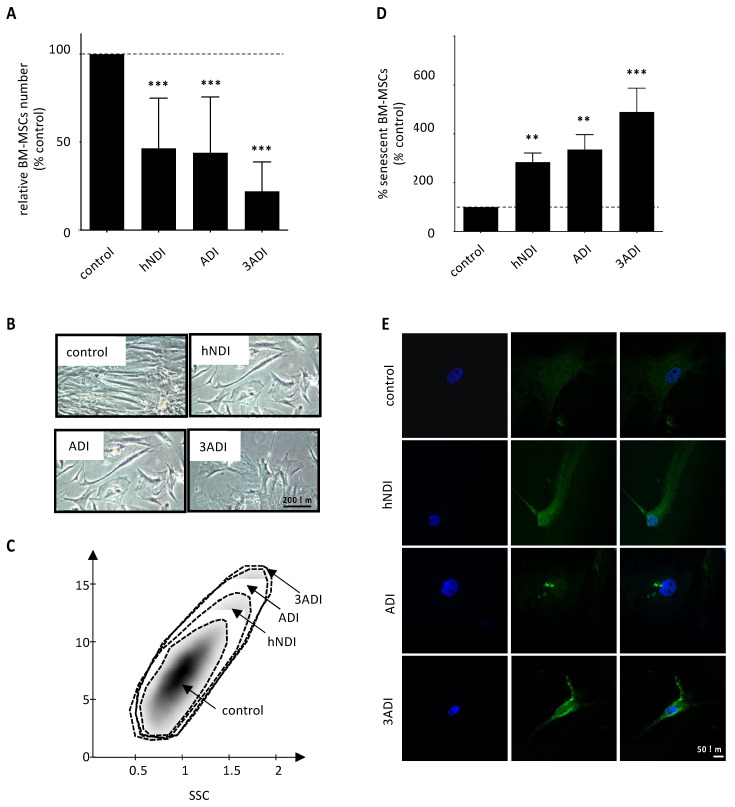
BM-MSCs enumeration and senescence are impacted after 21 days of pesticide exposure. (**A**) BM-MSCs enumeration (Trypan blue exclusion method) indicates a lower number of cells after exposure to pesticides (hNDI, ADI, 3ADI, *n* = 36) for 21 days in vitro. (**B**) Representative bright-field micrographs of BM-MSCs after 21 days of exposure and (**C**) representative forward-scatter (FCS) and side-scatter (SSC) flow cytometry plot, revealing abnormal cell morphology after pesticides exposure. (**D**) β-galactosidase activity in BM-MSCs indicates a higher number of positive cells (senescent cells) after pesticide exposure (*n* = 11), and (**E**) anti-β-Galactosidase immunostaining confirms these results. Data are expressed as percent of the control ± SEM; **, *p* < 0.001; ***, *p* < 0.0001; Friedman or Kruskall–Wallis followed by post hoc multiple comparisons test. Scalebars: 200 µm (**B**) and 50 µm (**E**).

A dose-dependent decrease in the amplification of viable normal BM-MSCs was observed after 21 days of culture continuously exposed in vitro to pesticides, as described previously [9,10]. At D21, untreated cells presented a major amplification (15,016 ± 4761 fold-increase). By comparison to this control condition (100%), the amplification rate was significantly lower after pesticide exposure (hNDI: 46 ± 4%, *p* < 0.0001; ADI: 44 ± 5%, *p* < 0.0001 and 3ADI: 22 ± 3%, *p* < 0.0001) (Figure 1A). This decreased amplification was associated with features of cell suffering, mainly at the 3ADI dose, as shown by bright field microscopy and forward-scatter and side-scatter flow cytometry plots (Figure 1B,C). Concomitantly, pesticides induced a significant dose-dependent increase in senescence (vs. control condition: hNDI: 2.9 ± 0.4 fold-increase, *p* = 0.0038; ADI: 3.4 ± 0.6 fold-increase, *p* = 0.0030; 3ADI: 4.9 ± 1.0 fold-increase, *p* < 0.0001) (Figure 1D,E). These results are in accordance with those published by our research consortium [9] and validate the experimental model and the chemical activities of pesticides used in the present study.

Maneb and mancozeb, the pesticides most frequently identified in food samples [8], are those for which the toxicity is the most documented [30,31]. These two compounds share the same chemical structure. Mancozeb is a combination of both maneb and zineb that contains 20% of Mn and 2.5% of Zn. Both maneb and mancozeb are degraded into a main organosulfur compound, ethylene thiourea (ETU: C3H6N2S), which is further degraded in ethylene urea and ethylene diamine. Consequently, their relative weight in the deleterious effect of the cocktail was determined in separate experiments by comparing the amplification rate of BM-MSCs after 21 days exposure to maneb alone, mancozeb alone and a cocktail with the five other pesticides (chlorpyrifos ethyl, dimethoate, diazinon, iprodione and imazalil). None of these three exposure conditions was able to recapitulate the effect observed with low doses of the complete mix, suggesting their additive and/or synergistic toxicity (Figure S1). Finally, HPLC analyses were performed to study the capacity of BM-MSC to metabolize maneb, mancozeb and diazinon, and these cells were not able to produce metabolites of interest (Figure S3).

**Figure 2:**
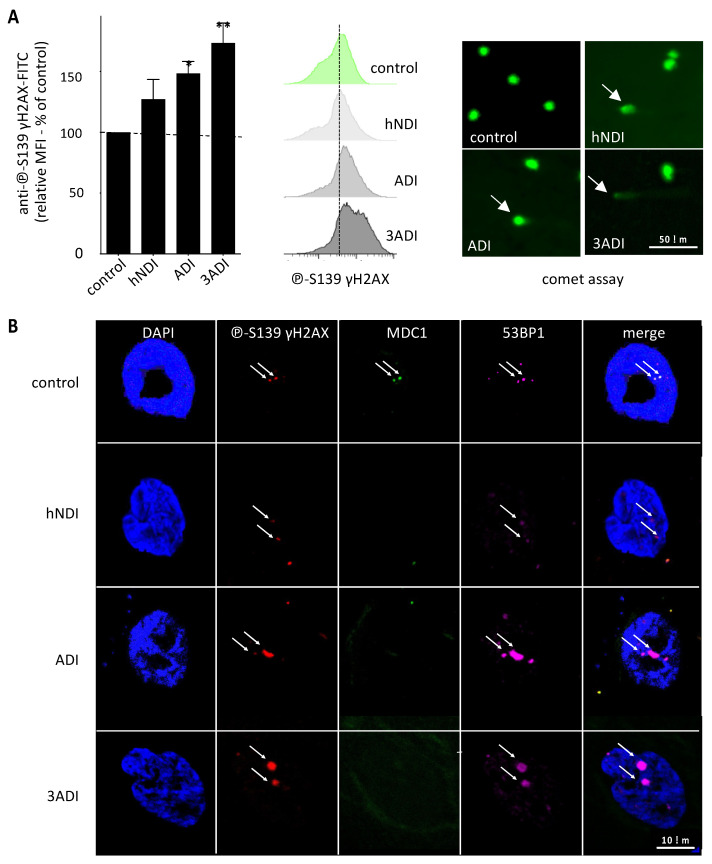
Pesticide exposure for 21 days induces DNA damage in normal BM-MSCs. (**A**) DNA damage with double strand breaks, quantified by ℗-S139-γH2AX immunoreactivity by flow cytometry, increases in BM-MSCs after pesticide exposure for 21 days (left panel, *n* = 6), as shown in a representative experiment (middle panel). Comet assays reveal increased nucleus DNA migration after exposure of BM-MSCs to pesticides for 21 days, as shown in a representative experiment (right panel). (**B**) ℗-S139 γH2AX, 53BP1, MDC1 triple immunostaining in BM-MSCs indicates a higher number of double positive 53BP1/℗-S139 γH2AX signals in the nucleus of exposed cells (FOCI) and a loss of MDC1 staining. Data are expressed as percent of the control ± SEM; *, *p* < 0.05; **, *p* < 0.01; Friedman followed by post hoc Dunn’s multiple comparisons test. Scalebar: 50 µm (**A**) and 10 µm (**B**).

The impact on DNA damage of the oxidative stress-induced senescence after exposure to the low-dose pesticide mix was evaluated by three different methods. The induction of DNA double strand breaks was appeared to be higher after pesticide exposure, by the dose-dependent increase in phospho-S139 γH2AX quantification by flow cytometry (vs. control condition: hNDI: 1.29 ± 0.2 fold-increase, NS; ADI: 2.15 ± 0.1 fold-increase, *p* = 0.0201; 3ADI: 1.74 ± 0.2 fold-increase, *p* = 0.0015) (Figure 2A). DNA fragmentation was confirmed by comet assay (Figure 2A). Finally, DNA integrity was evaluated by confocal microscopy studying the foci, focused on the recruitment of 53BP1 and MDC1 on the DNA damage sites identified by phospho-S139 γH2AX staining. In the control condition, the culture of BM-MSCs for 21 days induced a few small and discrete nuclear foci with adequate recruitment of 53BP1and MDC1. As presented in Figure 2B, whatever the dose, pesticide exposure clearly increased the number and size of foci. As expected, 53BP1 colocalized with phospho-S139 γH2AX, while a defective nuclear recruitment of MDC1 was observed. These results suggest that the double strand DNA breaks induced by pesticide exposure could be due to defective DNA repair.

### 3.2. Pesticide Cocktail Alters ALDH2 Expression and Activity, Inducing Acetaldehyde Accumulation in BM-MSCs

**Figure 3:**
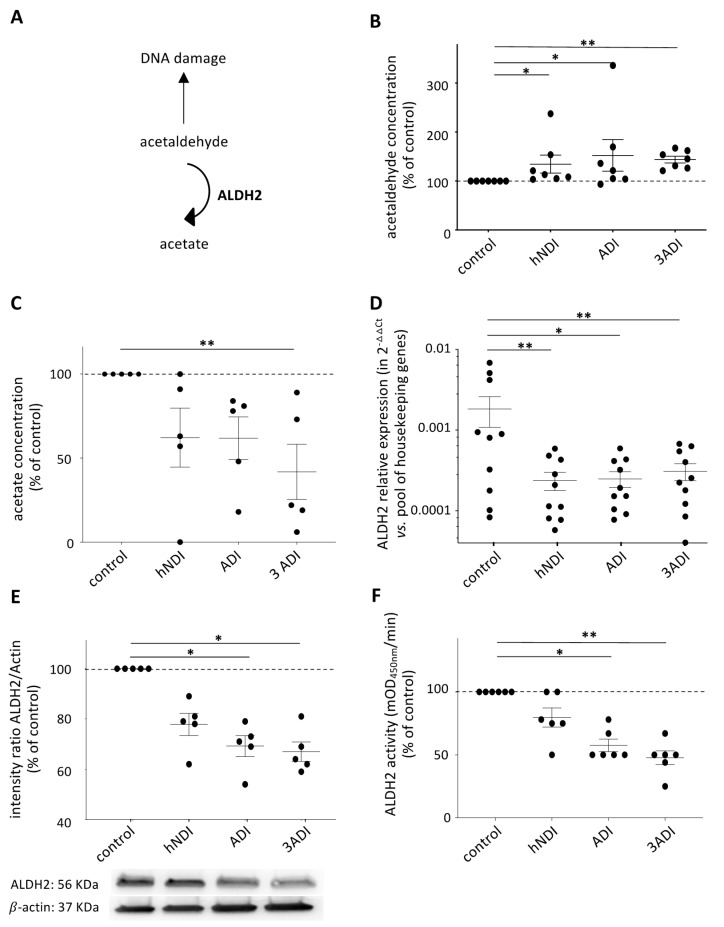
Mitochondrial acetaldehyde catabolism is altered after 21 days of exposure to pesticides. (**A**) Schematic representation of mitochondrial acetaldehyde catabolism via the ALDH2 enzyme. (**B**) Acetaldehyde (ALDH2 substrate) quantified by spectroscopy, is increased in exposed BM-MSCs (*n* = 7), and, concomitantly, (**C**) acetate concentration (ALDH2 product), is decreased in these cells (*n* = 5), indicating abnormal catabolism of acetaldehyde to acetate. (**D**) The analysis of ALDH2 relative expression indicates a strong decreased of ALDH2 mRNA abundance in case of exposure to pesticides (*n* = 10). (**E**) ALDH2 protein quantification by western blot also reveals a decreased ALDH2 production (*n* = 6). (**F**) Finally, ALDH2 enzymatic activity, quantified by spectroscopy, is decreased in BM-MSCs exposed to pesticides (*n* = 6). Data are expressed as percent of the control ± SEM; *, *p* < 0.05; **, *p* < 0.01; Friedman or Kruskall–Wallis followed by post hoc multiple comparisons test. The uncropped western blot images can be found in Figure S4.

DNA breaks result from various molecular mechanisms, especially from the cellular accumulation of such toxic metabolites as acetaldehyde (Figure 3A) [32]. Pesticide exposure significantly increased acetaldehyde levels in BM-MSCs (vs. control condition: hNDI: 1.4 ± 0.2 fold-increase, *p* = 0.0319; ADI: 1.5 ± 0.3 fold-increase, *p* = 0.0304; 3ADI: 1.4 ± 0.1 fold-increase, *p* = 0.0010) (Figure 3B). Acetaldehyde is detoxified in acetate [33] and, as expected, a concomitant decrease in acetate level was observed in the experimental conditions (Figure 3C). These results led us to focus on ALDH2 to explain the decrease in acetaldehyde catabolism.

*ALDH2* gene expression was quantified by RT-qPCR. Comparatively to the control condition (100%), pesticide exposure significantly decreased ALDH2 mRNA levels (hNDI: 31 ± 8%, *p* = 0.0030; ADI: 31 ± 1%, *p* = 0.0355; 3ADI: 31 ± 1%, *p* = 0.0015) (Figure 3D). The decrease in ALDH2 production was confirmed at the protein and functional levels (Figure 3E and Figure 3F, respectively). The other members of the ALDH superfamily (Table S3), known to be expressed in MSCs, were also quantified and their levels were not modified by pesticide exposure (Figure S2).

### 3.3. ALDH2 Inactivation by Disulfiram Induces Senescence and DNA Damage in BM-MSCs

**Figure 4:**
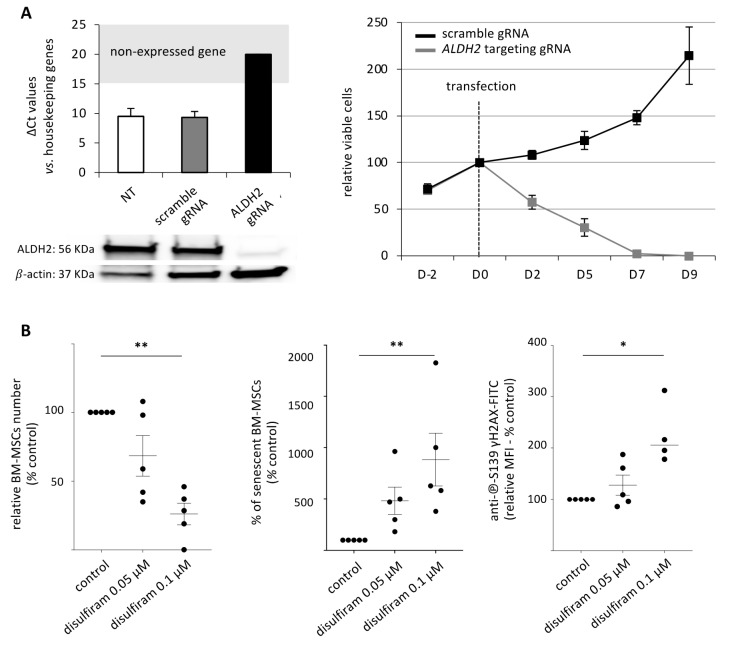
ALDH2 inhibition reproduces the consequences of pesticide exposure on BM-MSCs. (**A**) CrispR Cas9 based ALDH2 invalidation was checked by RT-qPCR and western blot analyses (left panel). ALDH2 invalidation in BM-MSCs induces cell death (right panel), ruling out the use of this strategy in the model. (**B**) Disulfiram, an inhibitor of ALDH2 activity, decreases the relative BM-MSCs number (*n* = 5), increases senescence (*n* = 4) and double strand DNA damage (*n* = 5), as quantified by anti-℗-S139 γH2AX immunoreactivity. Data are expressed as percent of the control ± SEM; *, *p* < 0.05; **, *p* < 0.01; Friedman followed by post hoc Dunn’s multiple comparisons test. The uncropped western blot images can be found in Figure S5.

The CrispR-Cas9 knock-out strategy was used to validate the implication of ALDH2 in the alterations of BM-MSCs observed after pesticide exposure, regarding proliferation, senescence and DNA damage. This strategy allowed efficient invalidation of *ALDH2* gene at the mRNA and protein levels (Figure 4A, left panel). This genetic modification induced an important lethality in BM-MSCs from D2 on (Figure 4A, right panel), impairing the use of this approach in the experimental system of pesticide exposure, ALDH2 being essential for cell survival [28].

Since the Crispr/Cas9 knockdown ALDH2 gene expression was not usable, a pharmacological inhibition strategy was then tested using disulfiram. At D14 of disulfiram exposure (0.1 µM), a significant decrease of viable BM-MSCs (26 ± 8% of the control condition, *p* = 0.0089; Figure 4B, left panel), increased senescence (883.5 ± 256% of the control condition, *p* = 0.0031; Figure 4B, middle panel) and increased DNA damage (125 ± 30% of the control condition, *p* = 0.0165, Figure 4B, right panel) were observed. These effects were initiated with lower doses of disulfiram (0.05 µM). Altogether, these results reinforce the role of ALDH2 involvement in the senescence and DNA damage processes observed after pesticide exposure.

### 3.4. Pesticide Exposure Alters the Functional Capacity of BM-MSCs to Support Primitive Hematopoiesis

**Figure 5:**
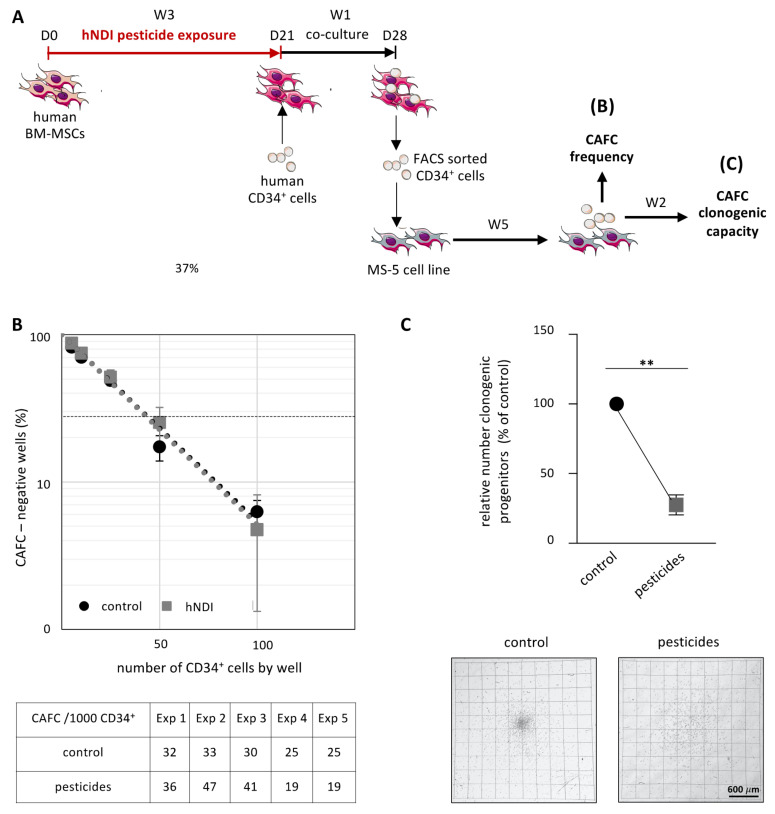
Exposure of BM-MSCs to pesticides alters the functionality of primitive hematopoietic progenitors. Human CD34^+^ cells were co-cultured for 7 days on BM-MSCs previously exposed or not for 21 days to low doses of pesticides (hNDI), and CAFC frequency was quantified by limiting dilution assays after 5 weeks on the MS-5 cell line. (**A**) Schematic representation of the experimental strategy. (**B**) Exposure of BM-MSCs to pesticides does not modify the frequency of CAFC in CD34^+^ cells (*n* = 5). (**C**) Exposure of BM-MSCs to pesticides induces a major decrease in the clonogenic capacity (CFU-GM, BFU-E and CFU-M) of CAFC (*n* = 5); representative morphological features of CFU-GM are shown in lower panel. Data are expressed as mean ± SEM; ** *p* < 0.01; Mann–Whitney test. Scalebar: 600 µm.

MSCs of the BM microenvironment play critical roles in primitive hematopoiesis to allow for the self-renewal and commitment of hematopoietic stem progenitor cells (HSPC). Their support of primitive hematopoiesis is classically well evaluated by CAFC limiting dilution assays [34]. The impact of low doses of pesticides on the microenvironment was consequently investigated, using human CD34^+^ HSPC cocultured for 1 week (D21 to D28) with BM-MSCs previously exposed or not (control condition) to low doses of pesticides (hNDI) for 21 days. On D28, CD34^+^ cells were FACS-sorted and then plated on the MS5 stromal cell line for 5 weeks to quantify CAFC frequency in CD34^+^ cells and their clonogenic capacity. Of note, pesticides were used exclusively during the 3 weeks of BM-MSCs expansion, before the co-culture with normal CD34^+^ cells. Consequently, normal hematopoietic cells were never directly exposed to them (Figure 5A). 

The frequency of CAFC in CD34^+^ cells was not modified by the exposure of BM-MSCs to pesticides (32 ± 6 CAFC/1000 CD34^+^ cells vs. 29 ± 2 CAFC/1000 CD34^+^ cells in the control condition) (Figure 5B). Nevertheless, previous pesticide exposure of the BM-MSCs altered their functionality, as shown by the 2.9-fold decrease of the CAFC clonogenic capacity (1.8 ± 0.6 clonogenic progenitors/CAFC, vs. 5.1 ± 1.3 clonogenic progenitors/CAFC in the control condition, *p* = 0.0003), associated with alterations in the size/morphology of the colonies generated (Figure 5C). Altogether, these results are in favor of an impairment of the maturation process of HSPC when in contact with BM-MSCs altered by low doses of pesticides.

**Figure 6:**
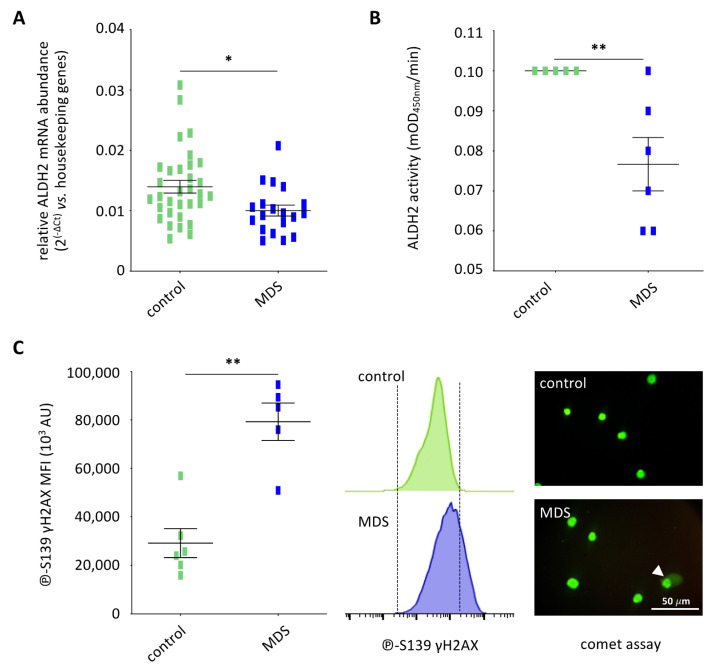
The ALDH2 pathway is altered in BM-MSCs from MDS patients. (**A**) ALDH2 expression is decreased in primitive MDS BM-MSCs, without in vitro exposure to pesticides (control BM-MSCs, *n* = 32; MDS BM-MSCs, *n* = 20). (**B**) ALDH2 activity is decreased in MDS BM-MSCs compared to control BM-MSCs (*n* = 6). (**C**) ℗-S139 γH2AX quantification by flow cytometry shows increased double strand breaks in MDS BM-MSCs compared to normal BM-MSCs (left panel, *n* = 6), as shown in a representative experiment (middle panel). A representative experiment shows an increased nucleus DNA migration in MDS-BM-MSCs compared to control BM-MSCs by comet assays (right panel). Data are expressed as mean ± SEM; *, *p* < 0.05; Mann–Whitney test. Scalebar: 50 µm.

Increased senescence of BM-MSCs and decreased HSPC clonogenic capacity are well established in patients suffering from MDS [13]. We thus focused on ALDH2 and DNA damage in primary BM-MSCs from MDS patients (Table S1), given our findings on pesticide exposure of such cells. When compared to BM-MSCs from healthy volunteers (100%), MDS BM-MSCs displayed a significantly lower ALDH2 expression (28 ± 5%, *p* = 0.0080) (Figure 6A) and enzymatic activity (76 ± 7%, *p* = 0.0152) (Figure 6B). Double strand breaks DNA were observed in these cells both by flow cytometry and comet assay, mimicking the results obtained after pesticide exposure of BM-MSCs from healthy volunteers (Figure 6C).

## 4. Discussion

The relationship between occupational exposure to high-dose pesticides and the occurrence of various diseases is well established, notably in hematological malignancies, as we recently reported in AML [4,6,35]. Moreover, the increasing use of pesticides since the 90′s has led to a contamination of water, fruits and vegetables, resulting in a daily exposure of the general population to residues from several pesticides [8]. The possible deleterious effects of low doses pesticides thus have to be studied. Our research consortium has already previously modelized in vitro what could be lifelong pesticide exposure, using a cocktail combining low doses of the seven pesticides frequently found in food and water by EFSA [8,9,10]. Three of them (diazinon, chlorpyrifos, mancozeb) have been reported to be associated with the occurrence of leukemia [36,37,38]. Here, we analyzed the in vitro effect of these low doses on primary BM-MSCs, focusing on potential DNA damage and their impact on primitive hematopoiesis control. The cocktail of pesticides used in our study (imidazole: imazalil/dithiocarbamates: maneb and mancozeb/dicarboximide: iprodione/organophosphates: chlorpyrifos ethyl, diazinon and dimethoate) is considered to be genotoxic [39,40,41,42,43,44,45] and classically associated with oxidative stress, an important cause of genotoxicity [46]. To date, no direct DNA binding has been reported for these pesticides. A direct DNA binding of pesticides was only described for organochlorinated pesticides, notably hexachlorocyclohexanes [47], which first bind to DNA bases via Van der Waals forces and halogen bonds. This induces an increase in helicity and base pair accumulation, as well as a more compacted structure of DNA which exposes more sites susceptible to promote DNA degradation through DNAse I.

Direct consequences of BM-MSCs exposure to pesticides, already reported, are dose-dependent senescence and decreased proliferation [9]. These results have been confirmed here, thus validating the experimental system. Low doses of pesticides promote an increase in reactive oxygen species (ROS) and stress-induced senescence in BM-MSCs [9] which can be explained by DNA damage, a well described consequence of exposure to pesticides [48,49,50]. Aiming to precise the incidence of pesticide cocktail on the machinery of DNA reparation, we analyzed foci (i.e., phospho-S139 γH2AX, 53BP1 and MDC1 colocalization) by confocal microscopy. This exposure of BM-MSCs promoted a lack of MDC1 recruitment in the foci, a feature reported to slow the mitosis process [51]. This could explain the decreased proliferation of exposed BM-MSCs. Of note, 53BP1 recruitment was not affected. The presence of 53BP1 in DNA damage foci might be explained by its binding to γH2AX in an MDC1-independent manner [52].

Acetaldehyde is known to induce ROS in various cell types, [53,54] and its accumulation in hematopoietic stem cells causes double strand DNA breaks [32]. The ALDH enzyme family is involved in the detoxification of aldehydes, playing a major role in cell survival, protection and differentiation, [55] and an interesting relationship has been established between ALDH and pesticides toxicity, notably mancozeb or maneb, in the pathophysiology of Parkinson’s and Alzheimer’s diseases [56,57]. The cellular acetaldehyde level is controlled by ALDH2, which detoxifies it into acetate [33]. The altered phenotype of BM-MSCs exposed to low dose pesticides that was observed here can be explained by the concomitant decrease in ALDH2 activity and increase in acetaldehyde levels in these cells. ALDH2 has been reported to be crucial in murine hematopoiesis [32,58]. In Asian populations, a functional ALDH2 polymorphism (ALDH2; rs671, Glu504Lys) is highly prevalent [59,60,61] and suspected to be related to an accelerated progression of bone marrow failure and malignant transformation in Fanconi anemia patients [62,63].

That ALDH2 deficiency in exposed BM-MSCs was shown here to impact their functional capacity to support primitive hematopoiesis. These cells did not alter the capacity of CD34^+^ to produce CAFC but the generation of clonogenic progenitors (BFU-E, CFU-M and CFU-GM) was severely decreased, demonstrating functional alterations of pretreated BM-MSCs in primitive hematopoiesis control. These results are consistent with data from mouse models where long-term exposure at high doses of agricultural pesticides decreased the proliferation and functional maturation of BM stem cells and hematopoietic progenitors [64,65]. Modifications in the clonogenic and differentiating capacities of hematopoietic progenitors have also been reported in mice exposed to low doses of a mix of 6 pesticides [66]. In humans, OPE to high doses of pesticides increases the risk of MDS [4], characterized by an ineffective hematopoiesis [67] and a reduced clonogenic capacity of HSPC [13]. Our study indicates that such reported in vivo observations could be explained, at least partly, by a primitive hematopoiesis supportive defect of the BM microenvironment exposed to low doses of pesticides. Our group recently published that about 40% of healthy subjects older than 50 years present clonal hematopoiesis [68]. The risk of developing a myeloid disorder (MDS, AML) by these subjects is increased but hopefully low, suggesting a multifactorial pathophysiology which may include environmental impact. This assumption is supported by the observation, in this study, of an underexpression of ALDH2 and an increase in acetaldehyde concentration in primary BM-MSCs from MDS patients. This suggests that normal BM-MSCs lifelong exposed to low-doses of pesticides may contribute to the pathogenesis of MDS. The hypothesis of a role of ALDH2 deficiency in such diseases is reinforced by a significantly younger median age at MDS diagnosis in Japanese than in western countries, [69,70] and by higher risk of AML after OPE in Asian population [6].

## 5. Conclusions

In conclusion, a new consequence of low-dose pesticide exposure on normal BM-MSCs is reported here, namely a decrease in ALDH2 enzyme production and activity. This induces an accumulation of cellular acetaldehyde, DNA damage and a decreased ability to support primitive hematopoiesis. Normal BM-MSCs exposed to pesticides and primary BM-MSCs from MDS patients present similar abnormalities, suggesting that a lifelong exposure to pesticide residues could promote the development of MDS through ALDH2 alterations in BM-MSCs.

This study has several limitations. The main limitation is the in vitro model with estimated theoretical concentrations in bone marrow cells. These results constitute a basis for future experimentations, notably in vivo experiments describing hematopoiesis in aged mice (at least 2 years) life-long exposed to low doses of the cocktail of seven pesticides. Finally, the decreased expression of ALDH2 in BM-MSCS from MDS patients leads us to hypothesize that this enzyme could be involved in MDS pathophysiology, without definitive conclusion. The relationship between pesticides exposure, ALDH2 decrease and MDS risk will require further biological and epidemiological studies.

## Data Availability

The data presented in this study are available on request from the corresponding author.

## Supplementary Materials

The following are available online at https://www.mdpi.com/article/10.3390/cancers13225699/s1. Figure S1: The cocktail effect of pesticides is required to induce a decrease in viable BM-MSCs, Figure S2: Pesticide cocktail exposure for 21 days does not modify aldehyde dehydrogenase-related genes expression in BM-MSCs, Figure S3: Quantification by HPLC of metabolites of maneb, mancozeb and diazinon, Figure S4: Uncropped western blot images for Figure 3, Figure S5: Uncropped western blot images for Figure 4, Table S1: Characteristics of MDS patients and healthy volunteers, Table S2: Doses of pesticides, Table S3: ALDH family characteristics.
