# Supplementary material for: Low-Dose Pesticides Alter Primary Human Bone Marrow Mesenchymal Stem/Stromal Cells through ALDH2 Inhibition
Source: Cancers (Basel). 2021 Nov 14;13(22):5699. doi: 10.3390/cancers13225699 (PMC8616329; doi:10.3390/cancers13225699)
Supplement: Supplementary file 1 [file cancers-13-05699-s001.zip › cancers-1408394-supplementary.pdf]

Supplementary Materials: Low-Dose Pesticides Alter Primary Human Bone Marrow Mesenchymal Stem/Stromal Cells through ALDH2 Inhibition

Amélie Foucault, Noémie Ravalet, Joevin Besombes, Frédéric Picou, Nathalie Gallay, Laetitia Corset, Jérôme Bourgeais, Sophie Hamard, Jorge Domenech, Pascal Loyer, Nicolas Vallet, Julien Lejeune, Emmanuel Gyan, Marie C. Béné, François Vallette, Christophe Olivier and Olivier Hérault

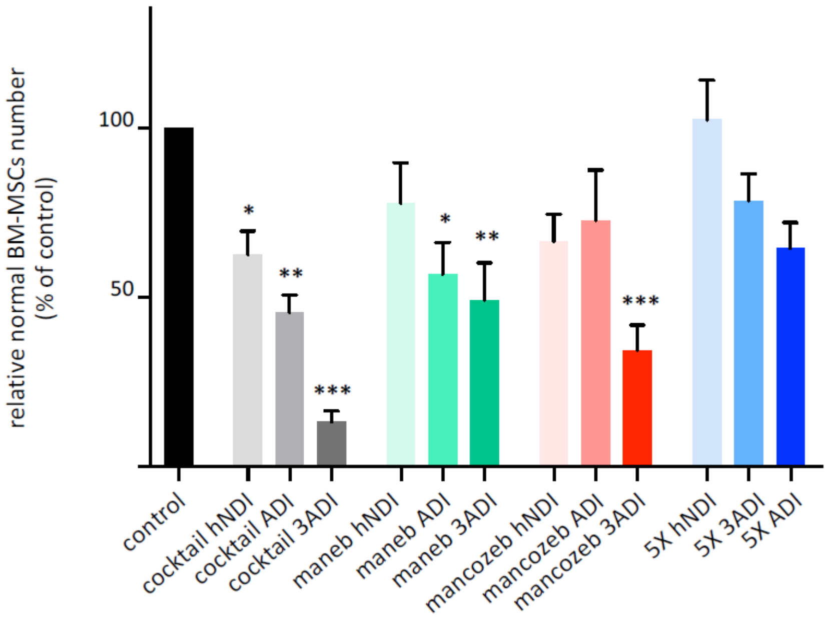

Figure S1. The cocktail effect of pesticides is required to induce a decrease in viable BMMSCs**.** Maneb alone, mancozeb alone and a mix of 5 other pesticides (iprodione, imazalil, chlorpyrifos ethyl, diazinon, dimethoate – cocktail named "5X") were used at hNDI, ADI and 3ADI. The effects obtained with the mix of these seven pesticides were recapitulated in neither of these 3 conditions. The number of BM-MSCs was moderately decreased when exposure to maneb, mancozeb or 5 other pesticides (5X hNDI, 5X ADI and 5X 3ADI), a major decrease being observed with high doses (3 ADI) of maned and mancozeb (n = 5).

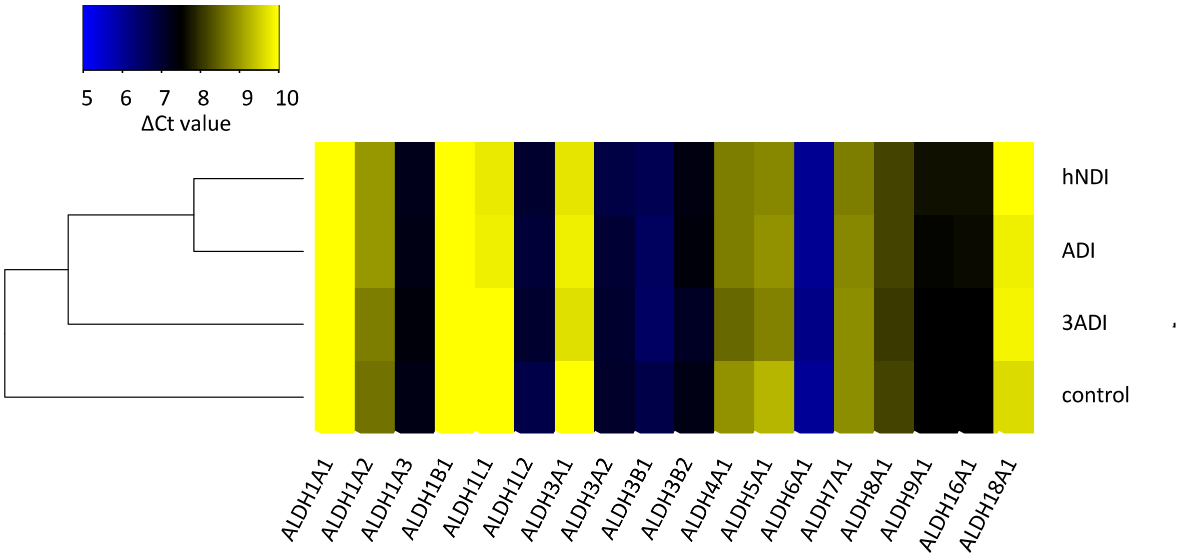

Figure S2. Pesticide cocktail exposure for 21 days does not modify aldehyde dehydrogenase-related genes expression in BM-MSCs. Heatmap showing different detectable isoforms of ALDH family genes (Table S3) in BM-MSCs after 21 days of pesticide exposure in vitro, (n = 10).

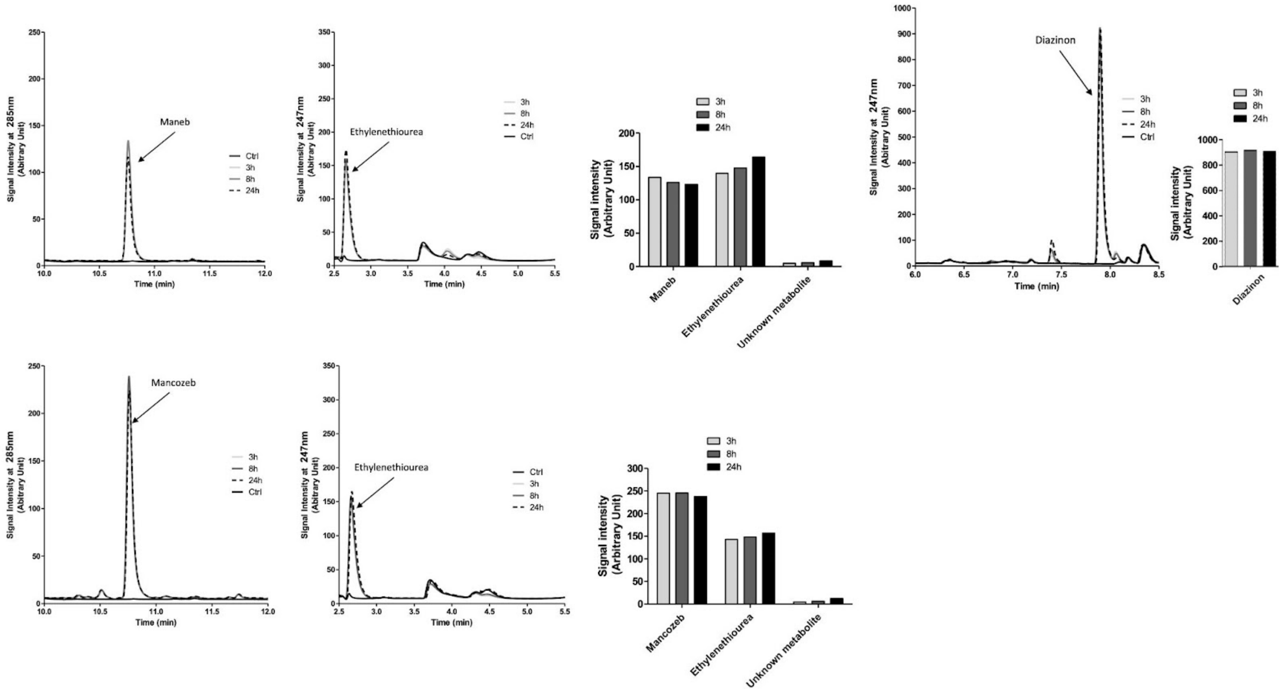

Figure S3. Quantification by HPLC of metabolites of maneb (upper left panel), mancozeb (lower left panel) and diazinon (right panel). BM-MSCs were exposed for 3, 8 and 24 h to 50 µM pesticide. The quantification of metabolites (ethylenethiourea and unknown compounds for maneb and mancozeb) indicates that the cells are not able to metabolize these pesticides.

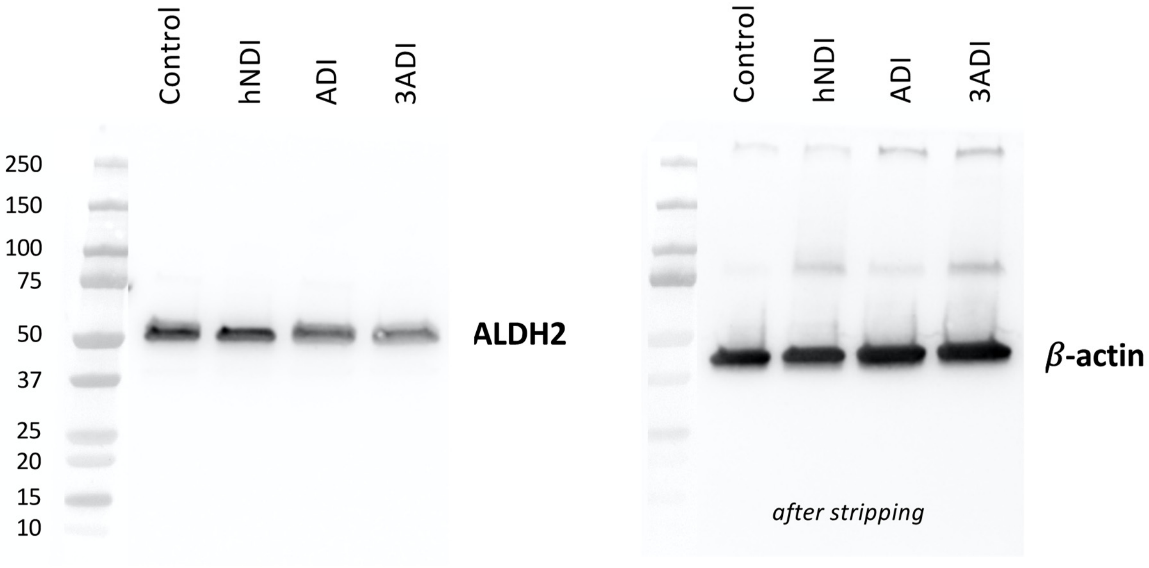

Figure S4. Uncropped western blot images for Figure 3.

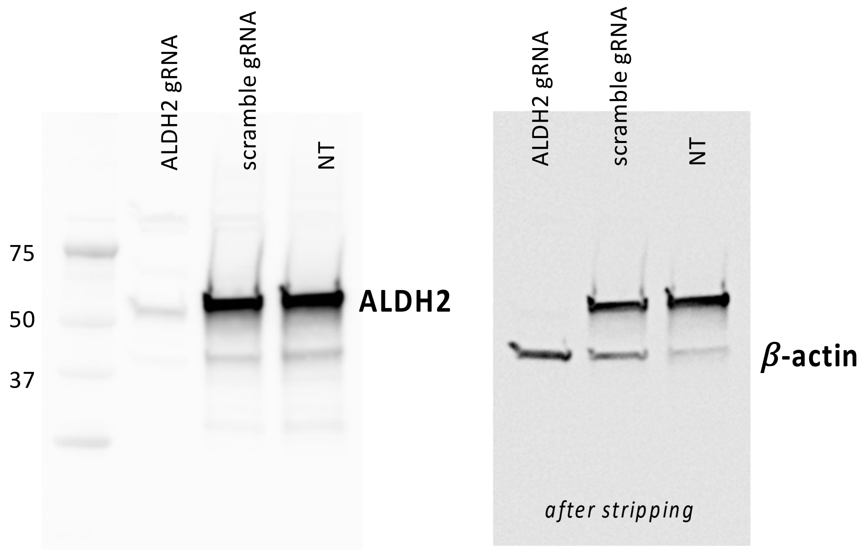

Figure S5. Uncropped western blot images for Figure 4.

Table S1. Characteristics of MDS patients and healthy volunteers.

MDS Patients	Age	Gender	Diagnosis	Cytogenetic	IPSS
MDS 1	72	F	MDS-EB1	46,XX,del(20q11)	0.5
MDS 2	69	M	MDS-MLD	46,XY	0.5
MDS 3	70	M	MDS-RS-SLD	46,XY	0
MDS 4	70	F	MDS-RS-SLD	46,XY	0
MDS 5	83	M	MDS-MLD	46,XY	0.5
MDS 6	70	M	LMMC	46,XY	0.5
MDS 7	69	F	MDS-MLD	46,XY	0
MDS 8	85	M	MDS-RS-SLD	46,XY	0
MDS 9	87	M	MDS-SLD	46,XY	0.5
MDS 10	77	M	MDS-MLD	45,XY, -7, del(5)(q13;q34), add(12)(p11)	1.5
MDS 11	86	F	MDS-MLD	46,XY	0
MDS 12	61	F	MDS-SLD	46,XX, 2 AC	0.5
MDS 13	94	M	LMMC	46,XY	NA
MDS 14	69	M	MDS-MLD	46,XY	0.5
MDS 15	82	F	MDS-SLD	46,XY	0.5
MDS 16	78	M	MDS-EB1	46,XY	2
MDS 17	76	M	MDS-SLD	46,XY	0
MDS 18	71	F	MDS-MLD	46,XY	0.5
**Healthy Volunteers**	**Age Mean (Range)**	**Male (Number)**		**Female (Number)**	
n = 39	58 (22–101)	21		18	

Table S2. Doses of pesticides.

Heading Title	Final Concentration μM		
	hNDI	ADI	3 ADI
chlorpyrifos ethyl	0.010	0.342	1.027
dimethoate	0.010	0.052	0.157
diazinon	0.003	0.008	0.024
iprodione	0.010	2.181	6.542
imazalil	0.070	1.009	3.028
maneb	0.730	2.262	6.785
mancozeb	0.720	2.251	6.754

Table S3. ALDH family characteristics.

ALDH Family	Subcellular Localisation	Chromosome	Preferred Substrate
ALDH1A1	cytosol	9q21.13	retinal
ALDH1A2	cytosol	15q22.1	retinal
ALDH1A3	cytosol	15q26.2	retinal
ALDH1B1	mitochondria	9q11.1	retinal and acetaldehyde
ALDH1L1	cytosol	3q21.2	10-Formyl tetrahydrofolate
ALDH1L2	unknown	12q23.3	unknown
ALDH2	mitochondria	12q24.2	acetaldehyde, 4-HNE and MDA
ALDH3A1	cytosol, nucleus	17p11.2	aromatic, aliphatic aldehydes
ALDH3A2	microsomes, peroxisomes	17p11.2	fatty aldehydes
ALDH3B1	cytosol	11q13.2	octanal
ALDH3B2	unknown	11q13.2	unknown
ALDH4A1	mitochondria	1p36.13	glutamate γ-semi-aldehyde
ALDH5A1	mitochondria	6p22.2	succinate semi-aldehyde
ALDH6A1	mitochondria	14q24.3	malonate semi-aldehyde
ALDH7A1	cytosol, nucleus, mitochondria	5q31	α-Aminoadipic semi-aldehyde
ALDH8A1	cytosol	6q23.2	retinal
ALDH9A1	cytosol	1q23.2	γ-aminobutyr-aldehyde
ALDH16A1	unknown	19q13.33	unknown
ALDH18A1	mitochondria	10q24.3	glutamic γ-semi-aldehyde
